# Malaria in South America: The Target is Close

**DOI:** 10.4269/ajtmh.16-0502

**Published:** 2016-09-07

**Authors:** Virgilio E. Failoc-Rojas, Carolina Molina-Ayasta

**Affiliations:** ^1^ Universidad Nacional Pedro Ruiz Gallo, Hospital Regional Lambayeque, Lambayeque, Peru. E-mail: virgiliofr@gmail.com; ^2^ Universidad San Martin de Porres, Lambayeque, Peru. E-mail: caritomolinaayasta@gmail.com

Dear Sir:

The article published by Quispe and others^1^ outlines Peru's three strategic plans to eliminate malaria in the Peruvian Amazon region. It is a significant achievement for Peru to implement a plan to eradicate malaria in the Amazon region, where the greater percentages of cases of malaria in Peru are concentrated.^2^

Malaria has devastating effects on the health and way of life of the people all over the world; however, it can be prevented and treated. In America, cases of malaria have been reduced by 58%, and malaria deaths have decreased by 70% since 2000.^3^ These figures are very encouraging and represent a starting point from which to propose new long-term goals for the eradication of malaria.

**Figure 1.**
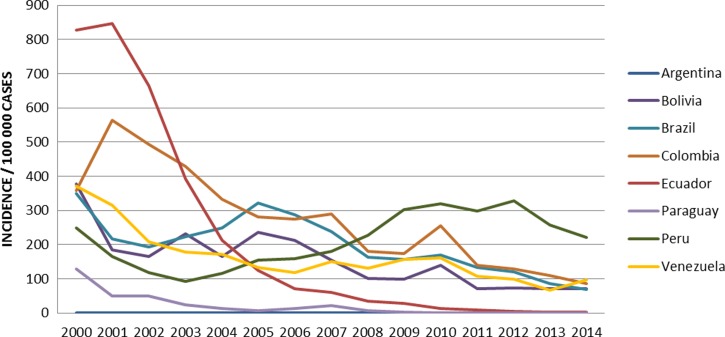
Incidence of malaria in Latin America 2000–2014.

To analyze the current situation in Peru compared with the rest of South America, we created a chart using World Health Organization data on cases of malaria and the total population, analyzing the incidences by year (Figure 1

). In South America (excluding Guyana, Suriname, and French Guyana), three countries (Argentina, Ecuador, and Paraguay) have achieved the preelimination phase. Peru appears to be the only country that has not been able to significantly reduce its cases of malaria, with 249.57 per 100,000 inhabitants in 2000 compared with 220.58 cases per 100,000 inhabitants in 2015, which indicates that Peru has neither kept up with nor applied the strategies used by its neighboring countries. Venezuela, Bolivia, and Brazil have gradually reduced their incidences of malaria during this period of time.

Multidisciplinary strategies^4^ for the control of malaria must be continued, from hygiene education for the community and better access to medication, to the creation of vaccines to interrupt the spread of malaria and the strengthening of the economic political strategies of malaria-endemic countries such as Peru.
